# Coral Thermal Tolerance: Tuning Gene Expression to Resist Thermal Stress

**DOI:** 10.1371/journal.pone.0050685

**Published:** 2012-11-30

**Authors:** Anthony J. Bellantuono, Camila Granados-Cifuentes, David J. Miller, Ove Hoegh-Guldberg, Mauricio Rodriguez-Lanetty

**Affiliations:** 1 Department of Biological Sciences, Florida International University, Miami, Florida, United States of America; 2 Department of Biology, University of Louisiana at Lafayette, Lafayette, Louisiana, United States of America; 3 Australian Research Council Centre of Excellence for Coral Reef Studies, James Cook University, Townsville, Queensland, Australia; 4 Comparative Genomics Centre, James Cook University, Townsville, Queensland, Australia; 5 Global Change Institute, University of Queensland, Saint Lucia, Queensland 4072, Australia; Leibniz Center for Tropical Marine Ecology, Germany

## Abstract

The acclimatization capacity of corals is a critical consideration in the persistence of coral reefs under stresses imposed by global climate change. The stress history of corals plays a role in subsequent response to heat stress, but the transcriptomic changes associated with these plastic changes have not been previously explored. In order to identify host transcriptomic changes associated with acquired thermal tolerance in the scleractinian coral *Acropora millepora*, corals preconditioned to a sub-lethal temperature of 3°C below bleaching threshold temperature were compared to both non-preconditioned corals and untreated controls using a cDNA microarray platform. After eight days of hyperthermal challenge, conditions under which non-preconditioned corals bleached and preconditioned corals (thermal-tolerant) maintained *Symbiodinium* density, a clear differentiation in the transcriptional profiles was revealed among the condition examined. Among these changes, nine differentially expressed genes separated preconditioned corals from non-preconditioned corals, with 42 genes differentially expressed between control and preconditioned treatments, and 70 genes between non-preconditioned corals and controls. Differentially expressed genes included components of an apoptotic signaling cascade, which suggest the inhibition of apoptosis in preconditioned corals. Additionally, *lectins* and genes involved in response to oxidative stress were also detected. One dominant pattern was the apparent tuning of gene expression observed between preconditioned and non-preconditioned treatments; that is, differences in expression magnitude were more apparent than differences in the identity of genes differentially expressed. Our work revealed a transcriptomic signature underlying the tolerance associated with coral thermal history, and suggests that understanding the molecular mechanisms behind physiological acclimatization would be critical for the modeling of reefs in impending climate change scenarios.

## Introduction

Coral reefs are of incredible value to human society, with a half billion people dependent on reefs which have been estimated to provide ecosystem services worth $375 billion per year [1], [2], [3]. However, this vast resource may be rapidly diminished by coral bleaching, a loss of the mutualistic intracellular dinoflagellates, *Symbiodinium*, and/or loss of photosynthetic pigments [4], originally described by Glynn in 1984 [5]. First reported in the 1870s [6], massive coral die-off from bleaching is expected to intensify as a result of increases in the magnitude and frequency of warm-water anomalies [7], [8], [9], the hyperthermal conditions responsible for bleaching. Therefore, the future of the reefs of the world is potentially in peril, with the potential for catastrophic coral bleaching and death resulting in the loss of half of the reefs worldwide in the next 20 to 40 years [8], [9], [10], [11]. Corals need to markedly increase their thermal tolerance at a rate of 0.2 to 1.0 °C per decade by adaptive or acclimative processes [10]. The exploration of physiological limits of corals and underlying molecular signatures is therefore of great importance in predicting the fate of corals in decades to come.

Current models of coral bleaching initiate with thermal- and photo-inactivation of *Symbiondinium* photosystem II and destruction of photosynthetic pigments by reactive oxygen species (ROS), proceeding to ROS-mediated host cellular damage and initiation of apoptotic pathways [9], [12], [13]. Multiple modes of dinoflagellate symbiont loss have been characterized, including the apoptosis and necrosis of host and symbiont cells [13], [14], [15], failure of host cell adhesion leading to detachment cells housing symbionts [16], exocytosis [17], and host-mediated autophagy [18].

Prior work on acquired hyperthermal tolerance in reef-building corals has largely focused on the potential for changes in dinoflagellate symbionts [19], [20], [21], [22], [23], [24], but a critical consideration in forecasts of the future of reefs as we know them is the role of thermal history and acclimatization to heat stress. Multiple studies have demonstrated the effect of thermal preconditioning on later bleaching susceptibility during natural heat stress events [25], [26], [27], [28], [29] or from experimental mesocosms [30], [31], [32], [33]. Maynard et al. [27] compared the 1998 and 2002 bleaching events on the Great Barrier Reef and found that there was a lower incidence of bleaching in 2002 even though there was higher solar irradiance in the latter event. Moreover, colony mortality in 1998 was not high enough to explain the result via different selection [27]. The effect of thermal preconditioning on subsequent heat stress has previously been demonstrated experimentally on *Acropora aspera* by Middlebrook et al. [32] in which 48-hour prestress treatments resulted in later resistance to bleaching temperatures, with no loss of symbionts, decrease in photopigments, or drop in quantum yield. Plastic responses to heat following differential histories of stress have been documented to occur even within a colony, in the case of *Goniastrea aspera* [34]. West faces of colonies suffered prior solar bleaching, which appeared to confer tolerance to heat stress as the west faces resisted bleaching during natural heat stress [34]. Subsequent work by Brown et al. [35] found less photoinhibition in symbionts of the west faces of colonies, along with higher expression of host superoxide dismutase and heat-shock proteins upon thermal challenge. Significantly, though, the response to climate change may be heterogenous across species [36].

There is an existing body of literature characterizing the molecular and cellular responses of several coral species to heat stress and bleaching. Gates et al. [16] found an induction of HSP70 after six hours of heat stress in *Montastraea franksi*, with a subsequent return to control levels with continued stress, followed by a later increase. DeSalvo et al. [37] explored the transcriptome of heat-stressed and bleaching *Montastraea faveolata*, finding differentially expressed genes with functions involving response to oxidative stress and HSP activity, calcium homeostasis, cell death, cytoskeletal structure, and metabolism. They propose a model in which ROS lead to the generation of reactive nitrogen species, disrupting calcium homeostasis, and with resultant changes in the cytoskeleton and calcification, cell adhesion, and the induction of cell death [37]. DeSalvo et al. [38] also queried the transcriptomic response of *Acropora palmata* and found similar themes across taxa, noting parallels between differentially expressed genes in response to heat stress in *M. faveolata* and *A. palmata*. Genes detected included those with putative roles in molecular chaperones, growth arrest, nucleic acid stabilization, elimination of damaged macromolecules, nitric oxide signaling, and actin cytoskeleton restructuring [38].

In our previous work [30], it was shown that preconditioning *Acropora millepora* for ten days to temperatures 3°C below bleaching threshold conferred thermal tolerance to the corals. This acquired bleaching resistance occurred with no detectable changes in either the *Symbiodinium* or bacterial communities, as shown by denaturing gradient gel electrophoresis [30]. Altogether, these pieces of evidence suggest that thermal prestress has a role in preventing later bleaching, conferring maintenance of *Symbiodinium* density. These prior results suggest physiological plasticity of one or more members of the coral holobiont (composed of the cnidarian host, *Symbiodinium*, and prokaryotes [39]) as the mechanism for resistance to bleaching. Our overarching question is whether corals will be able to acclimatize to rising ocean temperatures. To address this question, we asked what are the molecular-level effects that are associated with thermal tolerance, and how this response differs from that of thermal injury. This necessitates the exploration of the molecular underpinnings of thermal tolerance plasticity, as well as thermal injury associated with bleaching. The molecular response of the coral host in thermal-tolerant preconditioned coral holobionts has not been previously characterized. Here we examined the host transcriptomes of both thermal-tolerant and heat-sensitive corals. We also identified thermal preconditioning treatments effective in the rapid acquisition of thermal tolerance for *A. millepora*. We present the first evidence of the transcriptional response of the host associated with acquired thermal tolerance in *A. millepora*, along with the profile of thermal injury observed in non-preconditioned corals. Furthering the understanding of the response of corals to heat stress will provide information critical for the conservation of reefs as we know them. For instance, such knowledge will help determine whether corals are acclimatizing, and which corals have the capacity to do so at a rate compatible with their survival in a changing global environment. Genes of interest in acclimatization may be followed-up as potential targets of rapid evolution or epigenetic modification in response to global climate change, potentially answering questions regarding adaptive responses of corals to looming threats. The application of this mechanistic knowledge will prove practical in management plans for conservation of reefs, holding the potential to identify tolerant and at-risk reefs.

## Results

**Figure 1:**
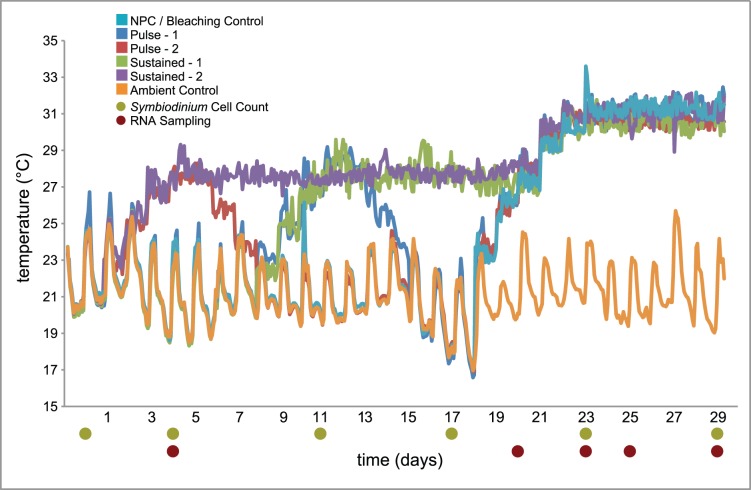
Temperature profiles of the thermal treatments to which *Acropora millepora* fragments were exposed. Non-preconditioned (NPC) treatment with no pre-stress period prior to exposure to 31°C. Pulse –1 (P1) treament was exposed to a 2-day 28°C pre-stress and returned to ambient temperature for 1 week prior to thermal challenge. Sustained –1 (S1) treatment was exposed to 10 days of 28°C prestress. Sustained –2 treatment was exposed to 14 days of 28°C prestress. Pulse –2 (P2) treament was exposed to a 2-day 28°C pre-stress and returned to ambient temperature for 2 weeks prior to thermal challenge. Sustained –2 treatment was exposed to 14 days of 28°C prestress. Ambient control (C) treatment was not challenged with any increase in temperature. This figure expands upon a smaller dataset originally published by Bellantuono et al. [30].

*A. millepora* coral fragments were exposed to preconditioning treatments, with details regarding the treatment of coral fragments available in the Materials and Methods section. In brief, control treatments (C) were treated only with ambient reef flat temperature water (17°C to 25°C). Sustained-1 treatment (S1) tanks were subjected to ten days of 28°C thermal preconditioning prior to a 31°C thermal challenge, while sustained-2 (S2) treatment was heated to 28°C for 17 days prior to exposure to 31°C thermal challenge. Pulse-1 (P1) and pulse-2 (P2) treatments were exposed to 28°C prestress for 48 hours one- and two weeks prior (respectively) to a 31°C thermal challenge. The non-preconditioned (NPC) treatment was ramped up directly from ambient temperature to thermal challenge temperature. Temperature log data is displayed in Fig. 1.

### *Symbiodinium* Density of Corals with and without Preconditioning

**Figure 2:**
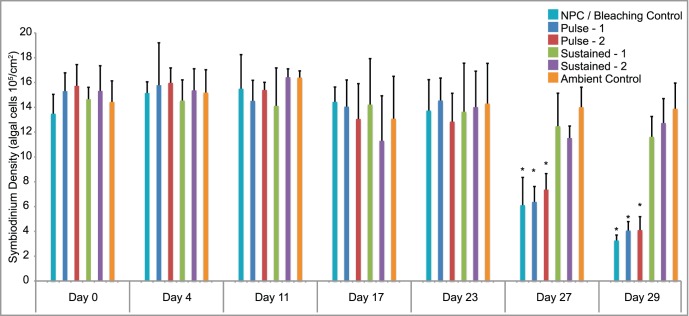
*Symbiodinium* density (algal cells per square centimeter). Resident *Symbiodinium* densities at 6 sampling times throughout the course of the experiment; days 23, 27, and 29 represent 2, 6, and 8 days of exposure to 31°C. Asterisks indicate group is significantly different from controls (p<0.001, one-way ANOVA with Tukey HSD, n = 4). A portion of the data presented in this figure was analyzed previously by Bellantuono et al. [30].

The objective of this work is to elucidate differences between heat-sensitive corals and those with acquired thermal tolerance, and we are using bleaching as an indicator of thermal injury. As such, *Symbiodinium* cell counts were used to quantitatively assess bleaching. In control nubbins exposed to ambient temperatures, *Symbiodinium* density was relatively constant throughout the course of the experiment, in the range of 1.3–1.6 algal cells 10^6^ cm^–2^. By day 29, after 8 days of exposure to water at 31°C, both coral nubbins that had not been exposed to thermal pre-conditioning (NPC) and those exposed to pulse treatments (P1 and P2) had suffered significant bleaching, *Symbiodinium* densities having decreased >70% (p<0.001, one-way ANOVA with Tukey HSD). By contrast, no significant declines in symbiont density were observed at that time in corals that had been subjected to sustained preconditioning treatments (S1 and S2) (p>0.20, one-way ANOVA with Tukey HSD; Fig. 2).

### Comparison of Gene Expression Levels

Our intent in applying microarray analyses was to shed light on the transcriptional differences between thermal tolerance and thermal injury. To investigate changes in gene expression associated with thermal tolerance, microarrays were used in a three way comparison between preconditioned (S1), non-preconditioned (NPC) and control (ambient) coral nubbins. Secondly, we explored changes in gene expression associated with thermal injury by comparing NPC and control corals. Note in reference to gene expression results, the terms *preconditioned*, *PC*, and *S1* collectively refer to the 10-day preconditioning treatment. The data discussed in this publication have been deposited in NCBI’s Gene Expression Omnibus [40] and are accessible through GEO Series accession number GSE41435 (http://www.ncbi.nlm.nih.gov/geo/query/acc.cgi?acc=GSE41435).

Our microarray analyses detected no differentially expressed genes (FDR-adjusted p<0.05) between treatments on Day 4 (18 days prior to thermal challenge, before any thermal manipulations of S1 or NPC corals) or Day 20 (preconditioned corals had been exposed to 28°C prestress for 10 days; meanwhile, non-preconditioned corals were also at 28°C en route to 31°C thermal challenge). ANOVA and pairwise comparisons of the microarray data identified differentially expressed genes (FDR-adjusted p<0.05) after two, four, and eight days of thermal challenge. The microarrays contained numerous redundant features, with many ESTs forming single contigs. All redundancies in our dataset were congruent, with gene expression trends in agreement.

**Figure 3:**
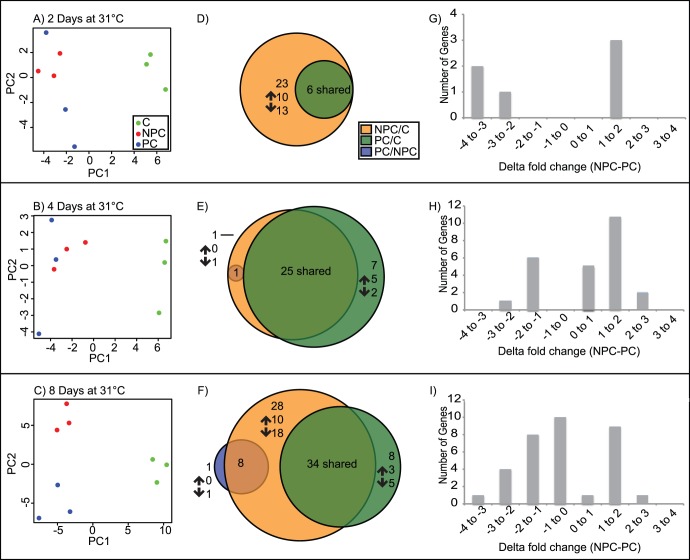
Microarray expression data. Rows A, B, and C represent gene expression from NPC, S1, and C treatments following 2, 4, and 8 days of 31°C, respectively. Left column contains principal component analysis plots of differentially expressed genes. Center column pie chart illustrated the number and trend of differentially expressed genes, with overlaps indicating differentially expressed genes detected across treatments. Column right indicates fold change differences between shared differentially expressed genes NPC and S1 treatments, both in reference to control.

At two days of 31°C thermal challenge, 23 genes were differentially expressed between non-preconditioned corals and control corals (10 and 13 up- and down-regulated, respectively), while six genes were differentially upregulated in S1 compared to controls (Fig. 3). At this sampling point, no differences between non-preconditioned and preconditioned treatments were detected by our analyses.

Still prior to visual signs of thermal bleaching and detectable symbiont loss, at four days of 31°C thermal challenge 27 genes were differentially expressed between non-preconditioned corals and control corals (18 up-, nine downregulated), 32 genes showed differences in preconditioned compared to controls (18 up-, 14 downregulated), and one gene was downregulated between preconditioned and non-preconditioned treatments (Fig. 3).

With eight days of thermal challenge at 31°C the non-preconditioned corals are exhibiting substantial bleaching, with the loss of nearly 80% of *Symbiodinium* (Fig. 2). At this point, 70 genes were differentially expressed in comparisons of non-preconditioned to control corals (23 up-, 47 downregulated), 42 differentially expressed between preconditioned and control (19 up-, 23 downregulated), and nine genes identified in the comparison of preconditioned to non-preconditioned treatments (four up-, five downregulated) (Fig. 3).

### Spatial Ordination of Gene Expression

PCA plots illustrate the spatial relationships of gene expression patterns amongst and between treatments (Fig. 3). The first principal component (PC1) separates preconditioned and non-preconditioned treatments from controls after two, four, and eight days of thermal challenge. After eight days of thermal challenge, when non-preconditioned corals are undergoing bleaching, non-preconditioned and preconditioned corals are distinctly different not just in their *Symbiodinium* density, but also in the ordination of their differential gene expression pattern, as illustrated by their separation on PC2 (Fig. 3c).

### Differentially Expressed Genes Shared Across Treatments

Following two days of 31°C thermal challenge, there is complete overlap in the identity of genes affected by non-preconditioned and preconditioned treatments during 31°C thermal challenge; all genes differentially expressed between S1 and Control are also differentially expressed between NPC and Control (Fig. 3, D–F). However, the overlap of genes involved does not illustrate the full picture, as though the same genes are affected, the magnitude of expression varies considerable (Fig. 3, G–I). There is a much more dramatic response from NPC/Control than from S1/Control both in the number of genes expressed, as well as the magnitude of expression.

After four days at 31°C, 74% (25 genes) of differentially expressed genes are shared between S1/Control and NPC/Control. The number of genes shared between NPC/C and PC/C continues to increases with time, with still more, 34 genes, shared after eight days of thermal challenge.

In all cases of shared, differentially expressed genes between NPC/Control and S1/control, the NPC/Control comparison has higher magnitude (in terms of absolute value) gene expression. The majority of shared genes differ by more than one-fold difference in expression (Fig. 3, G–I). The distinctions between treatments, initially shown by PCA of differentially expressed genes (Fig. 3), are borne out by differences in magnitude of expression, not by gene identity.

### Gene Ontology and Enrichment Tests

Blast2GO was used for annotation of EST contigs and to test for enrichment of gene ontology (GO) terms between pairwise comparisons (www.Blast2GO.org; [41]). Tests for enrichment of gene ontology terms found no significantly enrichment GO terms. As previously discussed, much of the differentiation between comparisons was in gene expression magnitude, not the presence or absence of different genes in the MAANOVA result. A test of GO enrichment is unable to elucidate this difference. Additionally, tests for enrichment are hampered by the lack of BLAST hits for 45% of the differentially expressed genes, precluding their inclusion in enrichment tests.

### Genes Involved in Thermal Injury

After eight days of thermal challenge at 31°C, the non-preconditioned corals bleached thoroughly. The differentially expressed genes between these non-preconditioned and control coral fragments illustrate the transcriptomic response of corals undergoing thermal injury. Our gene ontology analysis was informative for this comparison, with the 45 differentially expressed genes falling into GO IDs including response to oxidative stress, cellular homeostasis, and oxidation/reduction.

Non-preconditioned corals are characterized by a more extreme modulation of many of the same genes differentially expressed in preconditioned corals (Fig. 3). Notably, after eight days of thermal challenge these bleaching corals showed a marked increase in a *heme-binding protein 2-like* homolog, *permease*, *glycine-rich RNA binding protein*, *chorion peroxidase*, and a *mannose-binding lectin*. A decrease in transcripts was identified for homologs of a *mannose-binding lectin*, *ricin b lectin*, *CD151*, *universal stress protein*, N*F-κB inhibitor*, *calumenin*, *group II decarboxylase*, and *prefoldin 2*. It is important to distinguish that the up- and down-regulated *mannose-binding lectins* represent two distinct gene sequences.

### Gene Expression Co-occuring with Thermal Tolerance: Differential Expression between Preconditioned and Non-Preconditioned Treatments

The comparison of NPC to S1 transcriptome responses is important as it illustrates differences between bleaching, non-thermal tolerant corals and non-bleaching, thermal-tolerant individuals. At four days of thermal challenge at 31°C, no corals in the experiment were bleaching, but a single differentially expressed gene between NPC and S1 preconditioned treatments was detected. This gene, a *phosphate carrier protein* ortholog, is presumably involved in supplying inorganic phosphate to ATP synthase. Differential expression of phosphate carrier protein has previously been implicated in response to stress, as in the freeze tolerance of the wood frog *Rana sylvatica* [42].

After eight days of thermal challenge, several genes with stress-relevant ontologies differentiated the NPC treatment from S1. The 2.38-fold increased expression of a *mannose-binding lectin* in the preconditioned S1 corals over NPC is of great interest. The importance of lectins in symbiosis has been highlighted in previous work, including in adult *A. millepora* [43] and *Pocillopora damicornis* [44], as well as in the larvae of *Fungia scutaria* [45] and *A. millepora* [46], and in octocorals [47].

A putative *ferritin* ortholog had more than two-fold higher expression in NPC than in S1 corals. Ferritin is involved in response to oxidative stress, sequestering iron to prevent destructive Fenton reactions [48], [49]. *Transcription factor AP-1* exhibits higher expression in preconditioned corals. Among its diverse roles as a transcription factor acting in response to stimuli, AP-1 is involved in the gene regulatory response to stress [50].

### Gene Expression Co-occuring with Thermal Tolerance: Differential Expression between Preconditioned and Control Treatments

A complementary part of the thermal tolerance story includes changes that separate preconditioned corals and untreated controls from non-preconditioned corals and controls. All differentially expressed genes at two days of thermal challenge in the S1/Control comparison were also differentially expressed between NPC and control corals, but with distinct differences in trend (Fig. 3, G–1). While *lectin*, *tyrosine kinase receptor*, and *follistatin* homologs are upregulated in preconditioned corals in reference to controls, these genes are downregulated in non-preconditioned corals.

After fours days of thermal challenge, 32 genes are differentially expressed between S1 and control corals. This set of genes represents considerable overlap with the NPC/control comparison, but with much less change in magnitude, in all cases (Table 1). Two *heme-binding protein 2-like* orthologs are upregulated in preconditioned corals, as compared to controls. *Thymosin beta 4* exhibits slightly increased expression. Two genes coding for ribosomal proteins, *ribosomal protein l9* and *rbm3 protein*, show slightly decreased expression, with the ribosome-associated *nascent polypeptide-associated complex subunit alpha* also showing decreased expression.

**Table 1:** Differentially expressed genes at two, four, and eight days of thermal challenge.

2 days at 31°C				
Contig Name	Annotation (BLAST)	NPC-C	PC-C	PC-NPC
S_D021-H11_88	–NA–	2.11	0.21	
S_mge-C003-A11-pre80_T3	–NA–	1.85	0.49	
S_MGE-A050-C7-post50-T3	–NA–	1.22	0.15	
C_MGE-C019-A5-pre32_T3	tyrosine kinase receptor	−2.25	0.13	
C_D004-A11	lectin	−2.27	1.44	
C_D016-C4_27	follistatin	−2.52	1.01	
**4 days at 31°C**				
**Contig**	**Annotation (BLAST)**	**NPC-C**	**PC-C**	**PC-NPC**
C_D035-H1_8	heme-binding protein 2-like	3.07	0.85	
S_GS01WG04.b1.ab1	–NA–	2.26	0.15	
C_X001-E7_53	–NA–	2.10	0.52	
C_D037-C12_91	heme-binding protein 2-like	2.03	0.44	
C_D016-D12_92	glycine-rich rna binding protein	1.80	−0.10	
S_D021-H11_88	–NA–	1.78	0.19	
C_D040-B2_10	rbm3 protein	1.74	−0.14	
C_mge-A042-G6-post46-T	sodium potassium adenosine triphosphatase	1.54	0.58	
C_mge-A038-E12-post92-	coactosin-like protein	1.52	0.11	
C_MGE-B028-A11-prawn80	musashi homolog 2	1.50	0.11	
C_MGE-B015-H7-prawn55_	–NA–	1.38	0.33	
C_MGE-A027-C11-post82-	dynein light chain cytoplasmic	1.26	0.43	
C_D018-D12_92	nucleoside diphosphate kinase	1.01	−0.12	
C_MGE-B015-E7-prawn52_	heterogeneous nuclear ribonucleoprotein a2 b1 homolog	0.97	−0.31	
C_D012-F7_54	elongation factor 2	0.85	−0.48	
C_D017-G10_79	CDGSH iron-sulfur domain-containing protein 1	0.58	−0.33	
C_G031-E07.b1.ab1	ribosomal protein l9	0.51	−0.18	
C_mge-C011-B4-pre25_T3	nascent polypeptide-associated complex subunit alpha	0.41	−0.17	
C_MGE-B011-G7-prawn54-	solute carrier family 25 (mitochondrial carrier phosphate carrier) member 3	−0.11		−1.07
C_D010-C10_75	NF-κB inhibitor	−1.12	−0.11	
S_mge-C008-C2-pre10_T3	hypothetical protein DICPUDRAFT_79811 [Dictyostelium purpureum]	−1.22	0.21	
S_D010-A10_73	–NA–	−1.42	0.32	
C_mge-C011-F7-pre53_T3	thymosin beta 4	−1.66	0.11	
C_D023-A3_17	–NA–	−1.75	−0.26	
C_mge-A044-E12-post92-	calumenin	−1.95		
C_mge-A008-H1-4817-T3	decarboxylase	−1.99	−0.14	
C_mge-B023-E1-prawn4_T	group II decarboxylase	−2.27	−0.18	
C_D018-C8_59	elegans protein confirmed by transcript evidence		0.98	
S_MGE-A050-C7-post50-T3	–NA–		0.55	
C_D046-A9_65	electron transferring alpha polypeptide		0.50	
C_mge-A040-H12-post95-	–NA–		0.49	
S_GS01SG09.b1.ab1	–NA–		0.11	
S_mge-A041-F5-post37-T3	upf0687 protein c20orf27-like isoform 1		−0.31	
C_MGE-A032-H4-post31-T	chorion peroxidase		−0.48	
**8 days at 31°C**				
**Contig**	**Annotation (BLAST)**	**NPC-C**	**PC-C**	**PC-NPC**
C_D035-H1_8	heme-binding protein 2-like	3.02	1.41	
C_D018-C8_59	permease	2.71	1.15	
S_D021-H11_88	–NA–	2.62	0.43	
C_MGE-B015-H7-prawn55_	–NA–	1.84	0.29	
C_X001-E7_53	–NA–	1.71	0.70	
C_G028-C04.b1.ab1	mannose-binding lectin	1.59		
C_mge-C016-D4-pre27_T3	–NA–	1.40	0.16	
C_D016-D12_92	glycine-rich rna binding protein	1.15	0.10	
C_D003-E7	glutamine synthetase	1.12	0.10	
C_D012-A7_49	succinate- gdp- alpha subunit	1.05		
C_MGE-A032-H4-post31-T	chorion peroxidase or animal haem peroxidase	1.05		
S_mge-C003-F9-pre69_T3	–NA–	1.03	0.41	
C_mge-B017-G2-prawn14_	atp:adp antiporter	0.98	−0.12	
S_mge-A047-F6-post45-T3	–NA–	0.94		
C_MGE-A050-D1-post3-T3	potential c-type lectin (XP_002087457)	0.93	−0.24	
C_MGE-A020-E4-post28-T	fibrinogen-related domains	0.87		
C_MGE-A027-C11-post82-	dynein light chain cytoplasmic	0.82		
C_mge-C012-G9-pre70_T3	–NA–	0.78		
C_D049-C11_83	–NA–	0.26		
C_D028-B5_34	ferritin	0.25		−2.50
C_D027-D7_52	–NA–	0.23		
C_mge-C003-A1-pre0_T3	–NA–	0.23		0.71
S_D004-B9	UBX domain-containing protein 7	0.20		
C_mge-B035-C5-prawn34_	14-3-3 protein	−0.13		
S_D008-A9	ltv1 homolog	−0.13		−0.83
C_D009-C9	transcription factor ap-1	−0.18		2.38
C_MGE-C019-E2-pre12_T3	–NA–	−0.18		0.71
S_D030-E4_29	–NA–	−0.36		
C_mge-C001-G2-pre14_T3	ferritin	−0.40		
S_MGE-A018-E7-post52-T3	–NA–	−0.41		
C_mge-C004-H9-pre71_T3	ribosomal protein l37a	−0.43		−0.46
C_D011-C4_27	peroxiredoxin 6	−0.46		
C_mge-A036-H6-post47-T	–NA–	−0.48		−1.84
C_mge-C004-C8-pre58_T3	mitochondrial peroxiredoxin 5	−0.62	0.32	
C_G030-H02.b1.ab1	–NA–	−0.63		
S_mge-A040-C9-post66-T3	–NA–	−0.64		
C_MGE-A003-G11-postH18	CD151	−0.70		
C_D031-H1_8	tpa_inf: small cysteine-rich protein 1b	−0.72		
C_MGE-A015-H4-post31-T	predicted protein [Nematostella vectensis]	−0.73		
C_MGE-A009-C1-5772-T3	tpa_inf: small cysteine-rich protein 3	−0.77		
C_mge-C014-B7-pre49_T3	tpa_inf: small cysteine-rich protein 2	−0.83		
C_mge-B018-F3-prawn21_	predicted protein [Nematostella vectensis]	−0.87	−0.18	
C_MGE-A005-G11-19386-T	monooxygenase	−0.88		
S_D006-C11	sparc cwcv and kazal-like domains proteoglycan 2	−0.91	−0.44	
C_mge-C003-G2-pre14_T3	mannose-binding lectin	−0.93		2.38
S_mge-C008-C2-pre10_T3	hypothetical protein DICPUDRAFT_79811 [Dictyostelium purpureum]	−1.01	−0.12	
S_D030-H9_72	–NA–	−1.07	0.19	
C_mge-C007-F2-pre13_T3	myophilin	−1.08	−0.13	
C_D003-B10	protein NDRG3	−1.12	−0.21	
S_MGE-A014-E11-POST84-T3	NADPH-dependent fmn reductase	−1.16	−0.30	
S_MGE-A034-H6-post47-T3	–NA–	−1.17		
C_G031-E03.b1.ab1	universal stress protein (bacterial)	−1.19	−0.14	
C_mge-C011-F7-pre53_T3	thymosin beta 4	−1.29	−0.41	
C_MGE-A009-D7-57751-T3	ricin b lectin	−1.38		
C_D045-H5_40	A-macroglobulin receptor	−1.39	−0.44	
C_GS01XC11.b1.ab1	predicted protein [Nematostella vectensis]	−1.45		
C_D010-C10_75	NF-κB inhibitor	−1.45	−0.50	
C_G030-C08.b1.ab1	synaptic 2 or 3-oxo-5-alpha-steroid 4-dehydrogenase	−1.46	−0.25	
S_D019-D4_28	–NA–	−1.49	0.24	
S_MGE-C017-F11-pre85_T3	–NA–	−1.71	0.15	
S_D004-H4	–NA–	−1.85		
S_D022-E7_53	–NA–	−1.93	−0.34	
C_mge-A044-E12-post92-	calumenin	−2.02	0.46	
C_D046-E3_21	–NA–	−2.06		
S_D010-A10_73	–NA–	−2.06	0.46	
C_mge-A008-H1-4817-T3	group II decarboxylase	−2.13	−0.71	
C_D036-A1_1	Prefoldin 2	−2.13	−0.30	
C_GS01UH10.b1.ab1	mannose-binding lectin	−2.16	0.42	
S_D030-C2_11	–NA–	−2.52	−0.11	
C_D045-E9_69	–NA–	−3.82	−0.59	
S_D034-C10_75	rac serine threonine kinase		0.82	
C_mge-A038-E1-post4-T3	–NA–		0.58	
C_mge-C004-F10-pre77_T	oxidase peroxidase		0.51	
S_D011-G9_71	–NA–		−0.48	
S_D008-E3	–NA–		−0.69	
C_D023-A3_17	–NA–		−0.89	
S_D008-G9	zinc finger protein 704		−1.03	
C_D041-C3_19	tyrosine kinase		−1.19	
C_MGE-A049-H4-post31-T	cytoskeletal actin			−0.40

Differentially expressed genes detected via microarray analysis are represented by pairwise treatment comparison by day, indicating fold change difference for each treatment pair. Contigs from the microarray were identified using Blast2GO [41]; unknown genes are indicated by “–NA–.”

The nine genes detected as differentially expressed between S1/control and not between NPC and control after four days of thermal challenge potentially shed light on changes taking place prior to bleaching. One such gene is an *electron transferring alpha polypeptide* homolog, upregulated in the preconditioned treatment, with GO terms for this sequence including electron carrier activity, binding, and catalytic activity. A *chorion peroxidase* homolog is downregulated, with associated GO terms including response to stimulus, antioxidant activity, catalytic activity, and electron carrier activity.

After eight days of thermal challenge, 8 genes are unique to the S1/Control comparison. Among these, a *rac serine threonine kinase* homolog, with associated GO terms including signaling and response to stimulus, also showed increased expression. An upregulated sequence identified as an *oxidase peroxidase* by GO analysis has a potential role in antioxidant activity. Homologs of *zinc finger protein 704* and *tyrosine kinase* are both downregulated, with potential roles in DNA binding and catalytic activity, respectively.

### Genes Differentially Expressed Across Multiple Days

Though the majority of differentially expressed genes vary across days, several are detected at two or more sampling times. An mRNA putatively coding for a glycine-rich RNA binding protein was upregulated in NPC/C comparisons after two, four, and eight days of thermal stress. *Thymosin beta-4* shows decreased expression comparison of NPC/C on two, four, and eight days of 31°C thermal challenge, with the PC/C comparison showing a slight decrease after eight days of thermal challenge. *Calumenin* showed decreased expression in NPC/C comparisons over the course of thermal challenge, but displays an increase in preconditioned corals after eight days of thermal challenge. *NF-κB inhibitor* is downregulated after four and eight days in both NPC/C and PC/C comparisons, but to a much smaller degree in preconditioned corals than in non-preconditioned.

## Discussion

This is the first work to explore the transcriptional state associated with coral host thermal tolerance acquired by short-term preconditioning. A host molecular signature of bleaching resistance cements the role of the host as a critical factor in the persistence of the holobiont with impending threats of global climate change [51].

We have additionally shown that the duration of thermal preconditioning is critical for its efficacy. Middlebrook et al. [32] showed that *A. aspera* exposed to sub-bleaching preconditioning for 48 hours one- and two weeks prior to thermal challenge conferred resistance to bleaching and maintenance of thermal efficiency. However, our similarly-preconditioned pulse treatments (P1 and P2) were ineffective, bleaching alongside non-preconditioned corals, while sustained preconditioning (S1 and S2) led to thermal tolerance. These potential differences in effective preconditioning regimens between *A. millepora* and *A. aspera* bring attention to the consideration of physiological differences across species. Species-specific thermal physiologies are important considerations in the long-term management and modeling of coral reefs.

The effect of environmental stress on transcriptome states can be truly remarkable; for instance, in *Saccharomyces cerevisiae* more than half of the transcriptome is involved in response to environmental changes [52]. Intriguingly, the distantly-related *S. cerevisiae* and *Schizosaccharomyces pombe* exhibit a conserved stress response to most stress conditions, with upregulated genes involved in heat-shock, antioxidant roles, carbohydrate metabolism, and energy generation, and a downregulation in growth-related genes [52], [53], [54]. In *Drosophila melanogaster*, over 1200 genes were found to be differentially expressed in response to heat stress, and, while the specifics concerning stress responses in yeast and *Drosophila* differ, both involve common gene ontologies, including carbohydrate metabolism, cellular defense, protein folding, and energy production 55.

Prior investigation from our research group has been performed on the heat-stressed larvae of *A. millepora*, with transcriptome analysis performed using cDNA microarrays [46]. This work showed initial rapid induction of *heat shock proteins* in heat-stressed larvae, along with the decreased expression of a *fluorescent protein* and a *mannose-binding C-type lectin*. Curiously, these aposymbiotic larvae did not show detectable induction of genes involved in antioxidant stress response, suggesting that this stress may be associated with corals *in symbio* [46]. Vidal-Dupiol [44] identified the downregulation of a *mannose-binding C-type lectin* and a gene involved in calcium processes in *Pocillopora damicornis*. Using RNA-seq, Meyer et al. [56] also found increased expression of *heat shock proteins* with short-term heat stress, while observing decreased expression of *ribosomal proteins* and up-regulation of genes involved in ion transport and metabolism. Amongst these multiple studies, some common patterns fall out: initial upregulation of *heat shock proteins* in the first several hours of heat stress, then subsiding [16], [38], [46], with later changes occurring in *ribosomal protein* expression and calcium transport/homeostasis [37], [44], [56]. Also notably, *mannose-binding C-type lectins* show decreased expression in response to heat stress across disparate coral taxa [44], [46].

We propose a model of thermal tolerance in which the preconditioned coral host exhibits an attenuated transcriptional response, in comparison to the more extreme response in gene expression magnitude observed in non-preconditioned corals. It appears that acclimatization prior to thermal challenge prevents an extreme response in transcriptional magnitude, as indicated by the preponderance of co-differentially expressed genes between non-preconditioned/control and preconditioned/control comparisons, differing largely by magnitude of expression (Fig. 3 G–I).

Such drastic differences between non-preconditioned and preconditioned treatments (both in comparison to control) may represent compensation and repair on the part of damaged non-preconditioned coral. We may be observing a transcriptome overwhelmed. Notably, in this experiment, we were unable to detect changes occurring at 28°C. A dramatic stress, thermal challenge at 31°C, was required to produce detectable differential gene expression between treatments. The explanation for this could be either biological or technical; it could be indicative of the role of post-transcriptional gene regulation at lower levels of stress, or could represent technical limits of the experiment.

Many of the gene expression changes observed were of small magnitude, particularly in the preconditioned, thermal-tolerant corals. Small changes in gene expression have previously been shown to be of physiological relevance, as in the case of precocious sexual maturation in the brains of salmon [57]. In the case of handling stress on trout, it has been found that the majority of stress-response genes exhibit small or moderate changes in expression [58]. Acquired thermal tolerance via preconditioning may be a case of physiological fine-tuning on the part of the host, not massive transcriptional changes of large magnitude.

### *Lectins* Implicated in Thermal Tolerance

We detected the differential expression of several *lectins* over the course of the experiment (Table 1). Most strikingly, a *mannose-binding lectin* (C_mge-C003-G2-pre14_T3) was upregulated 2.83-fold in preconditioned corals after eight days of thermal challenge, compared to bleaching, non-preconditioned corals. *Lectins* have been shown to be critical in the recognition and onset of Cnidarian-algal symbioses, as in the work of Wood-Charlson et al. [45] on the coral *Fungia scutaria* and even earlier in *Hydra viridis* [59]. A mannose-binding lectin termed Millectin, isolated from *A. millepora*, has been show to bind to both *Symbiodinium* and pathogens [43]. Later on, Rodriguez-Lanetty et al. [46] showed that a homolog of Millectin in *A. millepora* larvae was down-regulated with thermal stress. Similarly, Vidal-Dupoil et al. [44] also identified a *mannose-binding lectin* in *Pocillopora damicornis* which is downregulated in association with thermal stress. Our results add to the body of work implicating lectins in the symbiosis, suggesting a role in thermal tolerance. The maintenance of a mannose-binding lectin may be important in the stability of coral-dinoflagellate symbiosis under duress.

### *Heme-binding Proteins*, *Ferritin*, and Iron-induced Oxidative Injury

*Heme-binding proteins* follow a pattern of expression in which they are upregulated in both non-preconditioned as well as preconditioned corals after four and eight days of thermal challenge. Though both experimental treatments show higher expression than controls, expression is generally higher in non-preconditioned treatments than in preconditioned treatments. After eight days of thermal challenge, *ferritin* expression was 2.50-fold higher in non-preconditioned corals than in the preconditioned treatment. These events may be indicative of response to iron-induced oxidative injury.

Superoxide formed by the breakdown of Photosystem II under heat stress and resultant damage to host mitochondria [13] is converted to hydrogen peroxide. If the resultant hydrogen peroxide is not processed by antioxidant systems, hydrogen peroxide can undergo iron-catalyzed cleavage to the extremely reactive hydroxyl radical [60]. This process, the Fenton reaction, can be circumvented by the sequestration of iron [60]. Both heme-binding proteins and ferritin can fulfill this role of iron sequestration [61]. As such, heme-binding proteins may be an important part of the response of corals to heat stress, as indicated by upregulation in both preconditioned corals, as well as in nonpreconditioned corals prior to and during bleaching.

Ferritins are involved in response to oxidative stress and in iron homeostasis [62]. *Ferritin* expression upregulation, in the case of our experiment, is associated with bleaching and not thermal tolerance, possibly indicating a loss of stasis and dramatic response on the part of the host. Differential expression of *ferritin* has previously been reported in several other experiments of coral heat stress [37], [46], [63], [64], [65]. Additionally, the work of Schwarz et al. [66] indicates that *ferritin* appears to be undergoing adaptive evolution in *A. millepora* and *A. palmata*.

### Transcription Factor AP-1, NF-κB inhibitor, and their Role in Apoptosis

The transcription factor AP-1 is a regulator of diverse cellular processes, including cell survival as well as death [67]. This gene, upregulated more than two-fold in preconditioned corals, may play a role in thermal tolerance.

Together, these two early response genes illustrate a hypothesis previously proposed using mammalian cells [68]. The early response genes comprising the AP-1 and NF-κB transcription factors are induced by environmental stress and thought to modulate responses to injury processes through the induction of target genes. Mattson et al. [68] showed that the DNA-binding of AP-1 and NF-κB are associated with changes in the cellular redox environment.

In one model of cnidarian bleaching, heat and light stress lead to hydrogen peroxide from the host and symbiont, as well as superoxide from damaged host mitochondria, causing the activation the transcription factor NF-κB [13]. NF-κB can also be activated by signals including p53 [69] and TNF-alpha [70]. NF-κB can then directly activate apoptotic processes, or cause the upregulation of nitric oxide synthases, initiating a cascade also culminating in apoptosis [13]. The work of DeSalvo et al. [38] supports the involvement of *NF-κB* in coral bleaching, detecting the upregulation of two *NF-κB p105* homologs in thermal stress experiments in *A. palmata*.

In mammalian cells, heat stress can affect the function NF-κB by inhibiting the translocation of NF-κB to the nucleus. This sequestration of NF-κB from the nucleus is believed to be facilitated by NF-κB inhibitor (IκBα), trapping NF-κB in the cytoplasm. Heat stress can both prevent the degradation of functional IκBα [71] and trigger an increase in mRNA expression of *IκBα* [72], [73].

By inhibiting NF-κB-mediated apoptosis and resultant bleaching in corals, NF-κB inhibitor has the potential to be a critical factor in host thermal tolerance and acclimatization. Our results suggest this, with *NF-κB inhibitor* expression lower in non-preconditioned corals than in preconditioned corals both prior to bleaching in non-preconditioned corals after four days of thermal stress, as well as while bleaching was underway, after eight days of thermal challenge.

From work on *A. millepora*, Pernice et al. [74] propose a model in which thermal stress activates caspase-3 dependent apoptosis in cells destined for destruction, with a concurrent increase in expression of an anti-apoptotic *Bcl-2* ortholog in surviving cells. Similarly, Kvitt et al. [75] identify a putative anti-apoptotic gene in *Stylophora pistillata*, *StyBcl-2*, coexpressed with a *caspase* during thermal stress.

By experimentally blocking the apoptotic pathway with a caspase inhibitor, Tchernov et al. [76] demonstrated the apparent protection from bleaching of thermally-challenged corals. We propose that the initiation of an inhibitor of NF-κB may similarly act to arrest the apoptotic cascade, preventing bleaching, as observed in preconditioned corals in this experiment.

### *Thymosin* as an Antioxidant and the Role of *Tyrosine Kinase Receptor* in Response to Oxidative Stress

Originally proposed to be a thymic hormone [77], thymosin beta-4 is the main actin sequestering protein in cells, preventing its polymerization [78]. It has other, diverse roles in cells, including cell proliferation and regeneration, and anti-inflammatory activities [77]. Recently, thymosin beta-4 has been experimentally shown to increase antioxidant and anti-apoptosis gene response in murine cells challenged with oxidative stress [79]. *Thymosin beta-4* shows decreased expression in non-preconditioned corals throughout the thermal challenge, with a slight decrease in expression in preconditioned corals only occurring after eight days of thermal challenge. Its role in corals is as-yet unknown, but it could potentially be involved in cell survival.

After two days of 31°C thermal challenge, a putative *tyrosine kinase receptor* was downregulated in the NPC treatment and slightly upregulated in the PC treatment, in comparison to controls. The occurrence of this differential expression prior to the onset of bleaching is suggestive of a potential regulatory role in symbiosis. Importantly, work in other systems has show that tyrosine kinase receptors respond to oxidants [80], [81], [82].

### *Calumenin* and Cnidarian/Dinoflagellate Symbiosis

Though the precise role of calumenin in cnidarian/dinoflagellate symbiosis has not been elucidated, *calumenin* is the most upregulated gene of the symbiotic state in *Anemonia viridis*, with multiple paralogs and cnidarian-specific duplications [83]. Additionally, *calumenin* is preferentially expressed in the endoderm of *A. viridis*, the tissue layer harboring dinoflagellate symbionts [83]. It is downregulated in NPC, decreasing in expression throughout thermal challenge (Table 1). In contrast, *calumenin* shows no significant decrease in expression in preconditioned corals, but is instead upregulated after eight days of thermal challenge, in comparison to controls (Table 1). The role of calumenin in symbiosis is unclear. Ganot et al. [83] suggest that calumenin is involved in host/symbiont recognition, through its regulation of *Sym32*. Calumenin belongs to the CREC protein family, a group of Ca^2+^-binding proteins with diverse cellular functions [84]. Previous work suggests the breakdown of a cellular calcium exclusion system as component of coral bleaching [85], [86], [87]. The upset of calcium homeostasis is also well-established as an apoptotic trigger [88], an important consideration given that apoptosis of host cells is one proposed mechanism of cnidarian bleaching [13]. Overexpression of *calumenin* in thermal-tolerant corals and decreased expression during bleaching may therefore be involved in host/symbiont signaling, calcium homeostasis, or in apoptosis.

### Absence of Differentially Expressed *hsp*s

This experiment did not detect differential expression of heat shock proteins in any treatments at any time point. Similarly, several studies examining thermal stress have not detected an upregulation of *hsp70* transcripts. Desalvo et al. [37] did not detect the upregulation *hsp70* after 24 hours of thermal stress in *M. faveolata*, while Mayfield et al. [89] also found no differential expression of *hsp70* in *Seriatopera hystrix* after 48 hours of heat stress. Voolstra et al. [65] identified no differentially expressed *hsps* after either 12 or 48 hours of heat stress in *M. faveolata*. It is possible that our sampling times following preconditioning and bleaching-threshold heat stress were not early enough to capture expression changes, as a heat shock protein transcriptional increase may have occurred but returned to normal levels in the 34.5 hour timespan between temperature increase and sampling. This interpretation is supported by Rodriguez-Lanetty et al. [46] in which transcriptional induction of *hsp70*, *hsp90*, *and gp96* in *A. millepora* larvae was detected after just three hours of exposure to heat. However, previous work on protein expression rather than mRNA has shown the rapid induction of heat shock proteins occurs in several corals species [16], [85], [90], [91].

Alternatively, biological variability leading to high variance between replicates may mask detection in this experiment. The differential expression of 488 unigenes between colonies in a common garden experiment with *A. millepora* calls attention to this potential explanation (Granados-Cifuentes et al. 2012, *in preparation*). Notably, *Hsp70* and *catalase* were among the differentially expressed genes (Granados-Cifuentes et al. 2012, *in preparation*); clearly, intercolony variability in gene expression must be a consideration, and may affect the detection ability of a thermal stress experiment.

Prior work in *M. faveloata* by Desalvo et al. [92] found that host transcriptomic states are associated with the type of symbiont occupying the host. This is not the case in the present work, as our previous work detected no shift in symbiont type [30], and sampling for the experiment at hand occurred in tandem with the aforementioned work.

Microarray results from samples collected Day 4 and Day 20 of the experiment revealed no differentially expressed genes. This is not a surprising result for Day 4, as no thermal manipulations occurred at that time on treatments assayed by microarray analysis. On Day 20, however, the S1 treatment had been preconditioned for 10 days and no changes in gene expression were detected. One potential explanation for this is that transcriptional changes during preconditioning were below the threshold of detection of the microarrays used for this experiment.

### The Importance of Understanding Acclimatization

An understanding of the physiology surrounding coral thermal history and associated tolerance is critical for the modeling of reefs in impending climate change scenarios. These projections will be invaluable in management strategies for the preservation of reefs. Biomarkers of coral health and stress have previously been developed (e.g. [93], [94], [95]), but markers of coral health from studies considering thermal history and indicative of resultant physiological plasticity must be implemented. This will allow the identification of at-risk, non-preconditioned coral populations for the enactment of management plans.

While phenotypic plasticity is in and of itself a critical piece of the capacity corals to cope with increasing environmental stressors, the interplay of differential gene expression and adaptation provides additional potential for the future of reefs. For instance, a transgenerational memory of stress has been shown in *Arabidopsis thaliana*, with the supposition that the genomic interactions of epigenetic processes may increase the likelihood of adaptation [96]. There is evidence that, in *Escherichia coli*, stress itself begets mutation, providing variation for natural selection to act upon [97]. Stress response genes tend to be associated with TATA boxes, with important repercussions [98]. TATA-containing genes tend to have a higher evolutionary lability, being more susceptible to mutation and regulated by more transcription factors than TATA-less genes [98].The elucidation of the interplay of stress, acclimatization and plasticity, and adaptation will become important under global climate change.

## Methods

### Coral Collection, Husbandry, and Thermal Stress Treatments

Collection of materials for downstream gene expression analysis was conducted in tandem with work reported in Bellantuono et al. [30], where temperature profile records are included. *A. millepora* branches 6–8 cm in length were cut from colonies on the reef flat in the vicinity of Heron Island (GBR), Queensland, Australia (23°33′S, 151°54′E) in June 2009. Colonies used for collection were previously genotyped for the presence of a carbonic anhydrase intron, and were confirmed to be of one type (Granados-Cifuentes et al. 2012, *in preparation*). Branches were embedded in marine epoxy in cut-off 15-ml centrifuge tubes. One-hundred fifty coral fragments for use in gene expression analysis and 168 fragments for assessing *Symbiondinium* density were allowed to recover for 20 days prior to the beginning of temperature manipulations.

The experiment was carried out in independently-heated 15 L tanks operated as open systems, receiving unfiltered seawater from nearby reef flat via a flowing seawater system at a rate of 0.3 to 0.4 liters/minute, with additional flow provided by 250 liter/hour submersible pumps. Temperatures manipulations tanks were controlled with independent heaters. Fragments were randomly assigned to one of six treatments, with four replicate tanks for each treatment. Each tank contained 16 coral fragments, originating from multiple colonies. Colony was not considered a factor in our experimental design. Control treatments (C) received ambient water (17°C to 25°C) with no temperature manipulation. The sustained-1 treatment (S1) tanks were heated to 28°C for ten days prior to being ramped up to 31°C. The sustained-2 (S2) treatment was heated to 28°C for 17 days prior to the increase to bleaching threshold. Pulse-1 (P1) and pulse-2 (P2) were heated to the prestress temperature for 48 hours one- and two weeks (respectively) prior to the ramp up to bleaching threshold temperature. The non-preconditioned (NPC) treatment was ramped up directly from ambient temperature to bleaching threshold temperature. Tank temperatures were ramped from 1–2°C per day, with temperature changes taking place at 06∶30. Ambient water temperature was a mean of 21.4°C (standard deviation = 1.6°C). The total length of the experiment was 29 days; the thermal challenge portion of the experiment comprised the final eight days with the final 8 days at bleaching threshold (mean bleaching treatment = 31.0°C, standard deviation = 0.6°C). The experimental system was covered with transparent plastic sheets during heavy precipitation. Tanks were covered with shade cloth from 11∶00–15∶00 daily to simulate light attenuation due to high tide and maintain temperature stability.

### *Symbiodinium* Density

To assess bleaching, coral fragments were collected from each treatment at 17∶00 on days 0, 4, 11, 17, 23, 27, and 29. One fragment was sampled from each tank replicate (n = 4). For the determination of *Symbiodinium* densities per surface area, cell counts were performed using a Neubauer improved haemocytometer (Hirschmann Laborgeräte), with coral area assessed by a wax coating method [99].

### RNA Extractions

One coral nubbin was collected at 17∶00 from each experimental and control tank for RNA extractions and immediately frozen in liquid nitrogen on Day 4 (18 days prior to thermal challenge), Day 20 (at which point preconditioned corals had been exposed to 28°C prestress for 10 days and non-preconditioned corals were also at 28°C en route to 31°C thermal challenge), and after two (Day 23), four (Day 25), and eight days (Day 29) of 31°C thermal challenge.

The topmost 0.5 cm of frozen coral nubbins were clipped and discarded using chilled bone cutters, and subsequently coral fragments ∼0.8 cm in length were cut. These fragments were crushed, and the frozen powder was transferred to Trizol Reagent (Invitrogen) and homogenized. Trizol RNA extraction protocol was followed as per manufacturer’s protocol through phase separation, at which point the aqueous layer was recovered by pipetting, gently mixed with an equal volume of absolute ethanol, and further cleaned with an RNeasy Mini kit (QIAGEN). RNA was quantitated using a NanoDrop ND-1000 UV-Vis Spectrophotometer (Nano-Drop Technologies), and integrity was assessed by electrophoresis on 1.25% MOPS-agarose gels (EmbiTec).

### Microarray Hybridization

Only RNA samples from control, NPC, and S1 were analyzed by microarray hybridization. These treatments were chosen as the S1 treatment (10 days of preconditioning at 28°C) exhibited acquired thermal tolerance, with non-preconditioned treatments providing for valid comparisons to corals with thermal injury, and controls allowing for comparison with corals not subjected to stress treatments. Three biological replicates of each treatment/sampling time combination were assayed. The cDNA microarrays implemented in experiments are third generation arrays for *A. millepora*, produced jointly by the Australian National University and James Cook University. Each microarray possesses 18,124 features, representing as many cDNA clones [100], [101]. Arrays for this experiment were manufactured in a single batch and randomly selected for each hybridization.

A reference design was chosen for this experiment due to its size and multiple treatments. RNA from all samples was mixed to make a reference sample. Complementary DNA was synthesized from 650 ng total RNA as per Array 900 kit protocol (Genisphere) using SuperScript III reverse transcriptase (Invitrogen). Reference cDNA samples were synthesized using primers for downstream capture by Cy3; experimental samples were synthesized using primers for downstream capture by Cy5. Hybridizations were performed with formamide-based hybribization buffer (Genisphere) under mSeries LifterSlips (Thermo Scientific). Arrays were prehybridized with 1 µg Human Cot-1 DNA for 90 minutes. Hybridization with cDNA was performed for 16 hours at 47°C. Arrays were washed in 65°C 2× SSC/0.2% SDS for 15 minutes, 2× SSC at room temperature for 15 minutes, and 0.2× SSC at room temperature for 15 minutes. Dye capture with Array 900 3DNA capture reagents (Genisphere) was performed at 50°C for 4 hours, using the aforementioned stringency washes. Following the final stringency washes, dried arrays were dipped in DyeSaver II (Genisphere). Immediately prior to scanning, each array was polished with a toluene/acetone solution (3∶1, v/v) and drying by centrifugation. Arrays were scanned on a GenePix Personal 4100A (Axon Instruments) microarray scanner; initial quality control, gridding, and raw data export were performed using GenePix Pro 4.1.

### Microarray Analysis

Data were quality-filtered and reduced to 5000 features in order to eliminate spots below the noise window. Background-subtracted mean intensity values were log- and lowess-transformed using R/Maanova version 1.18 [102]. A fixed-effect ANOVA model was fit to the normalized data. Empirical-Bayes Fs statistic [103] was used to test for differentially expressed genes at each sampling time. P-values for each clone were calculated from 500 permutations of residual shuffling. John Storey’s method for false discovery rate adjustment [104] was implemented, using an adjusted p-value threshold of less than 0.05. For pairwise comparisons, T-tests were performed within MAANOVA for the identification of significant interactions within sampling points, using a jsFDR-adjusted p-value cutoff of less than 0.05. To explore patterns present in the multidimensional gene expression data, principal component analysis (PCA) was performed using R version 2.10.0 [105]. Blast2GO was used to annotate genes and to test for enrichment of particular functional groups between treatments (www.Blast2GO.org; [41]).
