# Correction: Coral Thermal Tolerance: Tuning Gene Expression to Resist Thermal Stress

**DOI:** 10.1371/annotation/2b623dcd-9413-47b0-990d-c46c8e662ad4

**Published:** 2013-10-21

**Authors:** Anthony J. Bellantuono, Camila Granados-Cifuentes, David J. Miller, Ove Hoegh-Guldberg, Mauricio Rodriguez-Lanetty

In Table 1, data was omitted from the first section. Please see the corrected Table 1 here: 

**Figure:**
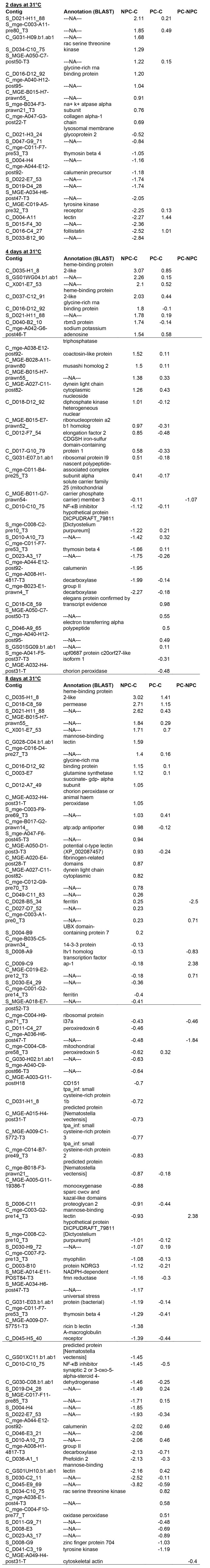


The email address for Corresponding Author Mauricio Rodriguez-Lanetty is missing. His complete email address is: rodmauri@fiu.edu

